# Impact of mature tertiary lymphoid structures on prognosis and therapeutic response of Epstein-Barr virus-associated gastric cancer patients

**DOI:** 10.3389/fimmu.2022.973085

**Published:** 2022-12-14

**Authors:** Yi-Xin Yin, Yi-Hong Ling, Xiao-Li Wei, Cai-Yun He, Bing-Zhi Wang, Chun-Fang Hu, Wen-Ping Lin, Run-Cong Nie, Jie-Wei Chen, Jin-Long Lin, Jie Zhou, Juan-Juan Xie, Jing-Ping Yun, Dan Xie, Li-Yan Xue, Mu-Yan Cai

**Affiliations:** ^1^ State Key Laboratory of Oncology in South China, Collaborative Innovation Center for Cancer Medicine, Sun Yat-sen University Cancer Center, Guangzhou, China; ^2^ Department of Pathology, Sun Yat-sen University Cancer Center, Guangzhou, China; ^3^ Department of Medical Oncology, Sun Yat-sen University Cancer Center, Guangzhou, China; ^4^ Department of Molecular Diagnostics, Sun Yat-sen University Cancer Center, Guangzhou, China; ^5^ Department of Pathology, National Cancer Center/National Clinical Research Center for Cancer/Cancer Hospital, Chinese Academy of Medical Sciences and Peking Union Medical College, Beijing, China; ^6^ Department of Hepatobiliary Oncology, Sun Yat-sen University Cancer Center, Center, Guangzhou, China; ^7^ Department of Surgery, Sun Yat-Sen University Cancer Center, Guangzhou, Guangzhou, China

**Keywords:** Epstein-Barr virus-associated gastric cancer, tertiary lymphoid structure, prognosis, therapeutic response, PD-1

## Abstract

**Background:**

Epstein-Barr virus-associated gastric cancer (EBVaGC) exhibits unique histological characteristics within the immune-cell-rich microenvironment, but the role of tertiary lymphoid structure (TLS) in EBVaGC is not yet fully understood.

**Methods:**

We retrospectively identified EBVaGC from 8517 consecutive GC cases from the two top cancer centers in China. Furthermore, we evaluated the prognostic value of TLS in 148 EBVaGC patients from our institute and then validated it in an external cohort (76 patients). TLS was quantified and its relationships with overall survival (OS) and therapeutic response were further analyzed. Multiplex immunofluorescence staining and targeted sequencing were used to characterize the composition of TLS and the genomic landscape, respectively.

**Results:**

In our study, EBVaGC was observed in 4.3% (190/4436) and 2.6% (109/4081) of GCs in the training and validation cohorts, respectively. TLS was identified in the intratumor (94.6%) and peritumor (77.0%) tissues with lymphoid aggregates, primary and secondary (i.e., mature TLSs) follicles in EBVaGC. Kaplan-Meier analysis showed that mature TLS in intratumoral tissues was associated with a favorable OS in the training and validation cohorts (p < 0.0001; p = 0.0108). Multivariate analyses demonstrated that intratumoral TLS maturation, pTNM, and PD-L1 expression were independent prognostic factors for OS (p < 0.05). Furthermore, the mature TLS was significantly associated with a good response to treatment in EBVaGC patients. Interestingly, the mutation frequency of SMARCA4 was significantly lower in the mature TLS groups.

**Conclusions:**

Intratumoral mature TLS was associated with a favorable prognosis and good therapeutic response, suggesting that it is a potential prognostic biomarker and predicts a good therapeutic response in EBVaGC patients.

## Introduction

Gastric cancer (GC) is the fifth most common cancer and the third leading cause of cancer-related deaths worldwide (1). Most GCs are typically diagnosed at an advanced stage with limited interventions available (1). What’s more, GC is a kind of tumor with strong spatial-temporal heterogeneity and complex tumor microenvironment, and advanced GCs present a significant heterogeneity in response to chemotherapy and immunotherapy. Thus, parameters that could describe and discriminate different tumor environments are urgently needed. Here we focus on the feature and prognostic value of the tertiary lymphoid structures (TLSs) in Epstein-Barr virus (EBV)-associated GCs (EBVaGCs) patients.

According to the molecular classifications of The Cancer Genome Atlas (TCGA), EBVaGC exhibits distinctive molecular profiles showing sensitivity to chemotherapy and immunotherapy (2–7). Besides, advanced EBVaGC presents a better response to chemotherapy with platinum and 5-fluorouracil (6, 8, 9). Furthermore, a considerable range of objective response rates (25%-100%) to PD-1 inhibitors has been shown in different studies of EBVaGC patients (10–15). Admittedly, the tumor microenvironment plays a very important role in tumors’ response to chemotherapy and immunotherapy. Thus, deciphering the heterogeneity of the tumor microenvironment is urgently needed for precise treatment (16).

TLSs are ectopic lymphoid aggregates that are typically formed in response to immunological stimuli, including solid tumors, and potentially act as a functional immune site (17). Recent studies have confirmed the merits of TLSs in response to anticancer treatment, such as chemotherapy, targeted therapy, and immunotherapy (18–23). The clinical implications of TLSs currently in EBVaGC patients remain to be elusive.

In this study, we comprehensively investigated the TLSs, PD-L1 expression, and the mutation profile in order to assess the prognostic value and analyze the clinicopathological relevance of TLSs in EBVaGC patients. We also sought to establish a promising clinicopathological prognostic model consisting of tumor microenvironment parameters in EBVaGC patients.

## Materials and methods

### Patient population

This study included 2 EBVaGC cohorts from 2 independent institutions in China. The SYSUCC cohort enrolled patients from Sun Yat-sen University Cancer Center (Guangzhou, China) from January 2015 to December 2019. The external validation cohort consisted of patients from the Chinese Academy of Medical Sciences and Peking Union Medical College (Beijing, China) from January 2014 to December 2017. Cases were collected retrospectively with the following eligibility criteria: (1) patients aged between 18 and 80 years; (2) pathologically confirmed diagnosis of GC; (3) positivity for EBV-encoded RNAs (EBERs); (4) tumor infiltration beyond the mucosa; (5) undergoing primary and curative resection; (6) provision of written informed consent; and (7) having complete follow-up information. The exclusion criteria were as follows: (1) a diagnosis or history of any other concurrent malignancies; (2) patients without available archived tumor tissues or complete follow-up data. Tumor differentiation was determined based on the World Health Organization (WHO) classification of Tumors of the Digestive System (2019 version). The tumor stage was determined according to the American Joint Committee on Cancer/International Union against Cancer TNM (tumor-node-metastasis) classification system (8th Edition). Approval for this study was granted by the Institute Research Medical Ethics Committee of Sun Yat-sen University Cancer Center and Chinese Academy of Medical Sciences and Peking Union Medical College.

### EBER *in situ* hybridization

EBER was examined on formalin-fixed paraffin-embedded specimens using an in situ hybridization (ISH) kit (ISH-7001, Zhongshan Jinqiao Biotechnology Co., Ltd., Beijing, China/Leica Biosystems, Newcastle, UK) strictly according to the manufacturer’s instructions. Positive and negative control tissues were synchronously included on each slide. The results were interpreted by senior pathologists to exclude nonspecific staining and misclassification.

### PD-L1 immunohistochemistry and scoring

The blocks were cut into 5-μm sections and processed for immunohistochemistry (IHC) using an anti-PD-L1 monoclonal antibody (22C3, Dako, Glostrup, Denmark) following a previously described protocol (24). Immunoreactivity for PD-L1 was scored using the combined positive score (CPS) method as follows: the number of positive cells (including cancer cells, lymphocytes, and macrophages)/the total number of surviving tumor cells, multiplied by 100. The positive expression of PD-L1 was defined as a CPS ≥ 1.

### Pathological review

All specimens were processed with hematoxylin and eosin (H&E) staining. Histopathological slides were assessed by two experienced pathologists (Y.-H. Ling and M.-Y. Cai), who were blinded to the patients’ outcomes. Both pathologists re-examined simultaneously the slides to solve the discrepancies with a double-headed microscope. TLSs were assessed on H&E slides, as previously described (25). Aggregates (Agg), primary follicles (FL-I), and secondary follicles (FL-II) were identified as the different stages of TLS development. Agg is characterized by loose, ill-defined clusters of lymphocytes. FL-I is characterized by oval-shaped clusters of lymphocytes. FL-II is defined by follicles with the formation of a germinal center and is considered as mature TLSs.

In the tumor center, tumors without any TLSs were defined as TLS- and others as TLS+. Cases included in the TLS+ group were further subdivided according to the maximum degree of TLS maturation. For example, FL-II: at least 1 FL-II regardless of the presence of Agg and FL-I (25). TLSs were classified as TLS-, Agg, FL-I, and FL-II according to this criterion. In the tumor margin (TM), TLSs were classified as TLSTM-, TM Agg, TM FL-I, and TM FL-II as in the tumor center. We also assessed the density of TM FL-II (i.e., mature TMTLSs) and FL-II (i.e., mature TLSs) per mm^2^. Areas of tumor margins located up to 2 mm from the infiltrative tumor border were scored in the analysis (25).

### Multiplex immunofluorescence staining and evaluation

Six cases with EBVaGC were subjected to multiplex immunofluorescence staining. The staining was performed according to the manufacturer’s protocol with the following markers: CD20 (Roche, 760-2531), CD21 (ZSGB-BIO, ZA-0525), CD4 (ZSGB-BIO, ZM-0418), CD8 (ZSGB-BIO, ZM-0508), and FOXP3 (Abcam, ab20034) using a PANO 7-plex IHC kit. Cell nuclei were stained with DAPI (1:2000 dilution). Multiplexed color slides were imaged with a Polaris slide scanner (AKOYA BIOSCIENCES) and five random areas on each sample were analyzed blindly at 200× magnification using HALO analysis software (Indica Labs).

### Targeted next-generation sequencing

To elucidate the genomic landscape of EBVaGC, 39 resected EBVaGC tumors were sequenced on a targeted panel consisting of 295 cancer-related genes (Burning Rock Biotech Ltd, Guangzhou, China), as previously described (26). In brief, DNA extraction and determination concentration were performed according to the manufacturer’s instructions. Hybridization, hybrid selection, and polymerase chain reaction amplification were then carried out, and the indexed samples were sequenced on a Nextseq 500 sequencer with paired-end reads. DNA translocation analysis was performed using both Tophat2 and Factera 1.4.3.

For TCGA (5), we extracted EBVaGC patients with pathological images (25 of 26 patients) and downloaded somatic mutation data (25 of 26 patients) and RNA-seq data (24 of 26 patients) from University of California Santa Cruz (UCSC) Xena database exploration program (https://xenabrowser.net/datapages/) for the comparative analysis.

### Immune infiltration analysis based on single-sample gene set enrichment analysis scores

The immune infiltration landscape of the TCGA GC samples was analyzed by single-sample gene set enrichment analysis (ssGSEA) according to the expression levels of immune cell-specific genes from the RNA-seq data. Marker genes representing 24 types of immune cells were defined with the recognized published article (27).

### Statistical analysis

Statistical analysis was computed using the SPSS 25.0 software package (Chicago, IL, USA) and R version 3.6.1. Categorical variables were analyzed by the chi-square test or Fisher’s exact test and continuous variables were analyzed with the t-test or Mann-Whitney test. For the univariate analysis, survival curves were obtained by the Kaplan-Meier method, and the differences between subgroups were analyzed by the log-rank test. Multivariate survival analyses were conducted using the Cox proportional hazard regression model. The curve was used to evaluate the accuracy of clinical risk factors in predicting overall survival (OS). Nomogram and calibration plots were constructed using the RMS package of R version 3.6.1. Mutation profiles and ssGSEA scores were performed using the maftools packages and gsva package. Two-sided *p* values of < 0.05 were considered statistically significant.

## Results

### Clinical characteristics

From January 2015 to December 2019, there were 4,436 patients diagnosed with GC, 190 (4.3%) of whom were identified as EBER-positive using ISH detection (**Figures S1 and S2**) in Sun Yat-sen University Cancer Center (Guangzhou). A total of 148 cases (SYSUCC cohort) with curative resection and complete clinicopathological data were included in the present study (**Figure S1**). Adjuvant chemotherapies with XELOX (oxaliplatin and capecitabine), SOX (oxaliplatin and S-1), or S-1 regimens were administered to 93 EBVaGC patients. In addition, eight patients received neoadjuvant chemotherapy with a XELOX or SOX regimen. In the external validation cohort, EBVaGC accounted for 2.6% (109/4081) of GCs, and 76 cases were included from January 2014 to December 2017. The baseline clinicopathological features are shown in **Table 1** and **S1**.

**Table 1:** Correlation between the maturation of TLS and clinico-pathological features in EBV-positive gastric cancer in the SYSUCC cohort.

Characteristics	Available number	Tumor center			Tumor margin			
		Non-mature TLSs	Mature TLSs	P value^a^	Non-mature TMTLSs	Mature TMTLSs	P value^a^
Gender	148			0.624			0.969
Male	131 (88.5%)	46 (35.1%)	85 (64.9%)		61 (46.6%)	70 (53.4%)	
Female	17 (11.5%)	7 (41.2%)	10 (58.8%)		8 (47.1%)	9 (52.9%)	
Age (years)	148			0.607			0.869
≤ 57.0^b^	74 (50.0%)	28 (37.8%)	46 (62.2%)		34 (45.9%)	40 (54.1%)	
> 57.0	74 (50.0%)	25 (33.8%)	49 (66.2%)		35 (47.3%)	39 (52.7%)	
Tumor size(cm)	148			< 0.001			0.005
≤ 4.75^c^	74 (50.0%)	14 (18.9%)	60 (81.1%)		26 (35.1%)	48 (64.9%)	
>4.75	74 (50.0%)	39 (52.7%)	35 (47.3%)		43 (58.1%)	31 (41.9%)	
Lauren type	145			0.302			0.643
Intestinal	43 (29.7%)	11 (25.6%)	32 (74.4%)		17 (39.5%)	26 (60.5%)	
Diffuse	21 (14.5%)	9 (42.9%)	12 (57.1%)		10 (47.6%)	11 (52.4%)	
Mixed	81 (55.8%)	30 (37.0%)	51 (63.0%)		39 (48.1%)	42 (51.9%)	
T stage	143			< 0.001			0.003
T1+T2	40 (28.0%)	2 (5.0%)	38 (95.0%)		10 (25.0%)	30 (75.0%)	
T3+T4	103 (72.0%)	46 (44.7%)	57 (55.3%)		54 (52.4%)	49 (47.6%)	
N stage	141			0.030			0.207
N0	37 (26.2%)	7 (18.9%)	30 (81.1%)		13(35.1%)	24 (64.9%)	
N+	104 (73.8%)	40 (38.5%)	64 (61.5%)		49 (47.1%)	55 (52.9%)	
M stage	148			< 0.001			< 0.001
M0	125 (84.5%)	33 (26.4%)	92 (73.6%)		48 (38.4%)	77 (61.6%)	
M1	23 (15.5%)	20 (87.0%)	3 (13.0%)		21 (91.3%)	2 (8.7%)	
pTNM	148			< 0.001			0.001
I-II	50 (33.8%)	6 (12.0%)	44 (88.0%)		14 4 (28.0%)	36 (72.0%)	
III-IV	98 (66.2%)	47 (48.0%)	51 (52.0%)		55 (56.1%)	43 (43.9%)	
Vascular invasion	142			< 0.001			0.006
Absent	68 (47.9%)	11 (16.2%)	57 (83.8%)		22 (32.4%)	46 (67.6%)	
Present	74 (52.1%)	36 (48.6%)	38 (51.4%)		41 (55.4%)	33 (44.6%)	
Neural invasion	142			0.013			0.306
Absent	47 (33.1%)	9 (19.1%)	38 (80.9%)		18 (38.3%)	29 (61.7%)	
Present	95 (66.9%)	38 (40.0%)	57 (60.0%)		45 (47.4%)	50 (52.6%)	
Differentiation	140			0.068			0.078
Poorly	83 (59.3%)	35 (42.2%)	48 (57.8%)		44 (53.0%)	39 (47.0%)	
Moderately	65 (46.4%)	18 (27.7%)	47 (72.3%)		25 (38.5%)	40 (61.5%)	
PD-L1 expression	148			0.090			0.435
CPS < 1	70 (47.3%)	30 (42.9%)	40 (57.1%)		35 (50.0%)	35 (50.0%)	
CPS ≥ 1	78 (52.7%))	23 (29.5%)	55 (70.5%)		34 (43.6%)	55 (56.4%)	
Recurrence	124			< 0.001			< 0.001
Yes	30 (24.2%)	21 (70.0%)	9 (30.0%)		20 (66.7%)	10 (33.3%)	
No	94 (75.8%)	14 (14.9%)	80 (85.1%)		29 (30.9%)	65 (69.1%)	
EBV-DNA	64			0.557			0.420
Pos	13 (21.5%)	6 (46.2%)	7 (53.8%)		5 (38.5%)	8 (61.5%)	
Neg	51 (78.5%)	19 (37.3%)	32 (62.7%)		26 (51.0%)	25 (49.0%)	

^a^Chi-square test; ^b^Median age; ^c^Median size. TLS, tertiary lymphoid structure; EBV, Epstein-Barr virus; PD-L1, programmed death-ligand 1; CPS, the combined positive score.

In the SYSUCC cohort, there were 131 (88.5%) males, with a median age of 57 years and a median tumor size of 4.75 cm. Most of the patients were diagnosed at stage III or IV (n = 98, 66.2%), and exhibited poor differentiation (n=83, 59.3%), vascular invasion (n=74, 52.1%), neural invasion (n=95, 66.9%), no recurrence (n=94, 75.8%), and positive PD-L1 expression (n=78, 52.7%). In the external validation cohort, there were 67 (88.2%) males, with a median age of 57 years and median tumor size of 4 cm. Most of the patients were diagnosed at stage I or II (n = 50, 65.8%), with poor differentiation (n=73, 96.1%), vascular invasion (n=49, 64.5%) and positive PD-L1 expression (n=63, 82.9%).

### Correlations between TLSs and clinicopathological characteristics

**Figure 1:**
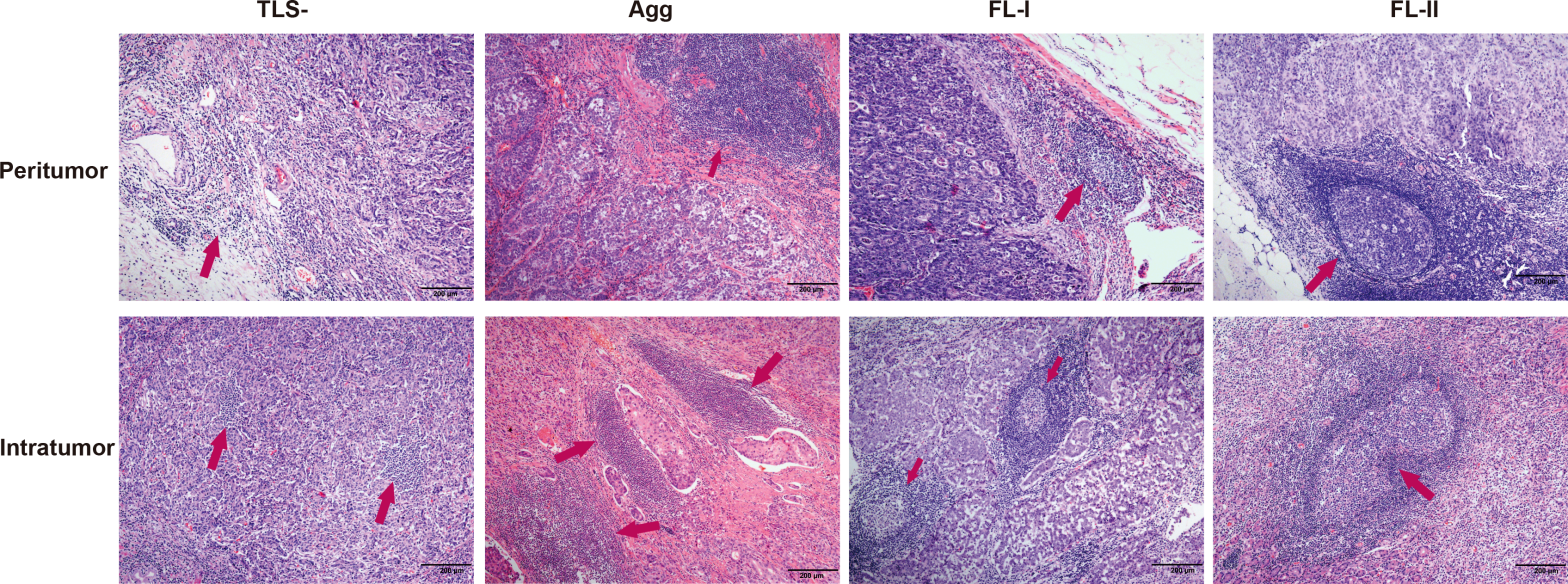
Histopathological classification of tertiary lymphoid structures. TLS-, without any TLSs (red arrow); Agg, Aggregates, loose, ill-defined clusters of lymphocytes (red arrow); FL-I, primary follicle, oval-shaped clusters of lymphocytes (red arrow); FL-II, secondary follicles, germinal center formation (red arrow).

In the SYSUCC cohort, 8 (5.4%) patients were TLS-, whereas 140 cases were TLS+. Among the TLS+ cases, the maximum degree of TLS maturation was Agg, FL-I, and FL-II in 15 (10.1%), 30 (20.3%), and 95 (64.2%) cases, respectively. Representative images depicting the degree of TLS maturation are shown in **Figure 1**. We further defined Agg, and FL-I as non-mature TLSs and FL-II as mature TLSs. Mature TLSs were negatively associated with tumor size (*p* < 0.001), lymphatic metastasis (*p* = 0.030), distant metastasis (*p* < 0.001), pTNM classification (*p* < 0.001), vascular invasion (*p* < 0.001), neural invasion (*p =* 0.013), and tumor recurrence (*P* < 0.001; **Table 1** and **Figure S3**). In the external validation cohort, 40 (52.6%) patients were non-mature TLSs, whereas 36 (47.4%) cases were mature TLSs. Mature TLSs patients were associated with earlier T category (*p* < 0.001), fewer lymphatic metastasis (*p* < 0.001), and earlier pTNM classification (*p* < 0.001; **Table S1**).

In the tumor margin, TLSTM-, TLSTM Agg, TLSTM FL-I, and TLSTM FL-II (**Figure 1**) were observed in 34 (23.0%), 11 (7.4%), 24 (16.2%), and 79 cases (53.4%), respectively. Consistent with the definitions at the tumor center, we determined TLSTM-, TM Agg, and TM FL-I as non-mature TMTLSs, and TM FL-II as the mature TMTLSs. In the external validation cohort, non-mature TMTLSs and mature TMTLSs were observed in 13 (17.1%) and 63 (82.9%) cases, respectively. Similar to the observations at the tumor center, further correlation analysis indicated that the mature TMTLSs were negatively associated with certain clinic-pathological features, including tumor size (*p* = 0.005), T category (*p* = 0.003), distant metastasis (*p* < 0.001), pTNM stage (*p* = 0.001), vascular invasion (*p* = 0.006), and tumor recurrence (*p* < 0.001, **Table 1** and **Figure S3**). Similarly, the association between TLSs and clinicopathological characteristics in the validation cohort was shown in **Table S1**.

### Multiplex immunofluorescence revealed the TLS structure

**Figure 2:**
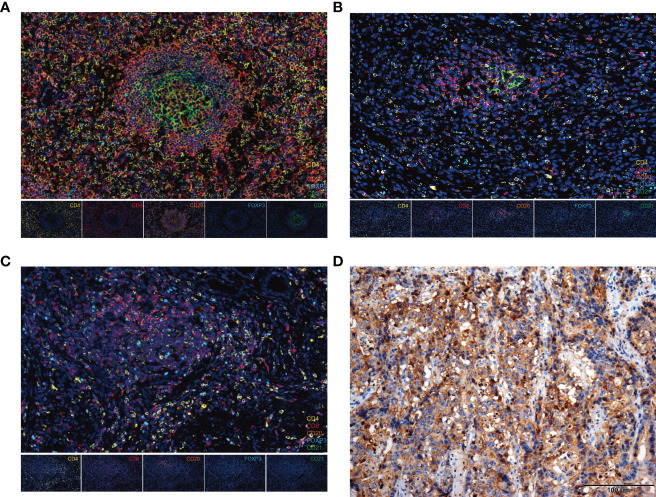
The composition of immune cells in TLSs and PD-L1 expression in EBVaGC tissues. **(A-C)** Representative images from multiplex immunofluorescence staining of EBVaGC tumor tissues showing TLSs (FL-II, FL-I, Agg) with the following markers: CD4, CD8, CD20, CD21, FOXP3, and DAPI. Magnification, x200. **(D)** PD-L1 expression by immunohistochemistry. EBVaGC, Epstein-Barr virus associated gastric cancer; TLSs, tertiary lymphoid structures; Agg, aggregates; FL-I, primary follicles; FL-II, secondary follicles.

To further characterize the immune cell subsets of the TLSs in our cohort, CD20^+^, CD4^+,^ CD8^+^, CD21^+^, and FOXP3^+^ cells were detected in the non-mature or mature TLSs tumor samples using multiplex immunofluorescence methods (**Figure 2**) (28). Tumor-infiltrating CD4^+^, CD8^+^, FOXP3^+^ T cells, CD20^+^ B cells, and CD21^+^ follicular dendritic cells (FDCs), are indispensable for forming the immune microenvironment (29–31). Of note, the percentage of infiltrating immune cells was significantly higher in the mature TLS than in non-mature TLSs (89.9% vs. 58.1%, *p =* 0.010; **Figure S4A**). In the mature TLSs samples, CD20^+^ B cells were the most frequent immune cells, followed by CD4^+^ T cells, CD8^+^ T cells, CD21^+^ FDCs, and FOXP3^+^ T cells at 34.37%, 27.90%, 25.27%, 9.27%, and 3.19%, respectively (**Figure S4B**), while their proportions in non-mature TLSs were 25.11%, 36.81%, 25.27%, 0.37%, and 12.44%, respectively (**Figure S4B**). Compared with non-mature TLSs, the proportion of CD21^+^ FDCs in the mature TLSs was significantly increased, while there were no significant differences in CD20^+^ B cells, CD4^+^ T cells, or CD8^+^ T cells. However, there was a higher infiltration of CD4^+^ T cells, CD8^+^ T cells, CD20^+^ B cells, and CD21^+^ FDCs in mature TLSs patients than in non-mature TLSs patients (*p* = 0.0001; *p* = 0.0004; *p* < 0.0001; *p* < 0.0001, respectively; **Figure S4C**). Moreover, the number of FOXP3^+^ T cells was lower in the mature TLSs than in the non-mature TLSs (*p* = 0.049, **Figure S4C**). Taken together, these results suggested that the increased infiltration of immune cells in mature TLSs may induce an immune-responsive microenvironment.

### Prognostic value of TLSs in EBVaGC

**Figure 3:**
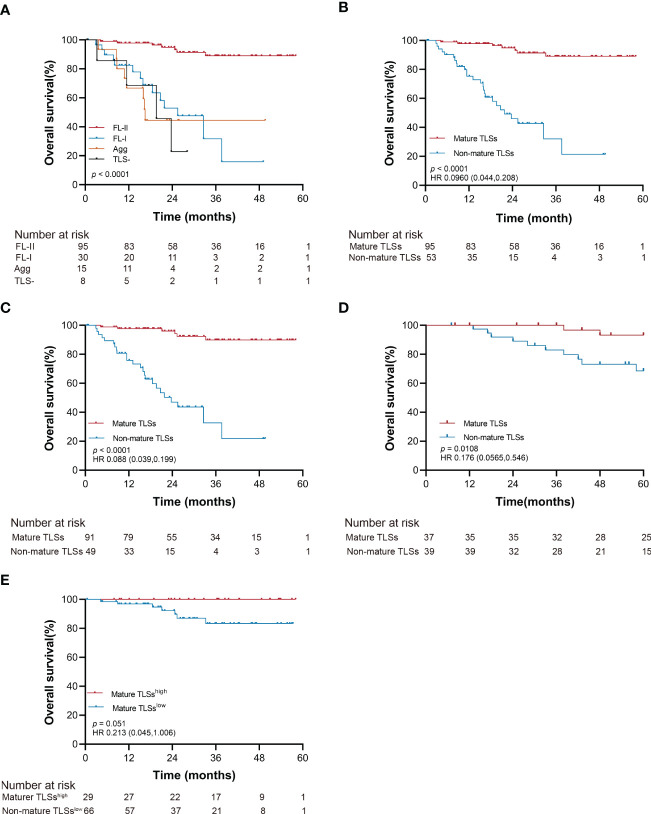
Kaplan-Meier estimates of overall survival according to the maturation of intratumoral TLSs (log-rank test). **(A)** Probability of survival of patients in TLS-, Agg, FL-I, and FL-II groups in the SYSUCC cohort. **(B)** Probability of survival of patients in mature TLSs and non-mature TLSs groups in the SYSUCC cohort. **(C)** Probability of survival of patients in mature TLSs and non-mature TLSs groups in the SYSUCC cohort, except the patients receiving neoadjuvant chemotherapy patients. **(D)** Probability of survival of patients in mature TLSs and non-mature TLSs groups in the validation cohort. **(E)** Probability of survival of patients in mature TLSs^high^ and mature TLSs^low^ groups in the SYSUCC cohort. EBVaGC, Epstein-Barr virus associated gastric cancer; TLSs, tertiary lymphoid structures; Agg, Aggregates; FL-I, primary follicles; FL-II, secondary follicles.

In the univariate analysis, tumor size (*p* < 0.001), vascular invasion (*p* < 0.001), neural invasion (*p* = 0.005), PD-L1 expression (*p* < 0.001), T category (*p* =0.007), lymph node status (*p* = 0.029), distant metastasis (*p* < 0.001), pTNM stage (*p* = 0.002), peritumoral TLSs maturation (*p* < 0.001), and intratumoral TLSs maturation (*p* < 0.001) were significantly associated with the overall survival of EBVaGC patients (**Table S2**; **Figures 3** and **S5**).

The OS improved to a statistically significant degree in the groups with mature TLSs or mature TMTLSs, compared with those without mature TLSs or mature TMTLSs (both *p* < 0.0001; **Figures 3A, B** and **S5A, B**). Among patients not receiving neoadjuvant chemotherapy, there was still a correlation between mature TLSs or TMTLSs and better prognosis (both *p* < 0.0001; **Figures 3C** and **S5C**).

In the validation cohort, the mature TLSs were also associated with longer OS (*p* = 0.0108; **Figure 3D**). However, there was no difference between the mature TMTLSs and OS (**Figure S5D**).

To determine the prognostic significance of TLS density in our cohort, we defined a threshold for separating patients with high and low TLS densities using R software. Our results showed that OS improved in patients with higher mature TLS density in the tumor center (mature TLS^high^, > 1.848 TLSs/mm^2^) (*p* = 0.051; **Figure 3E**). In the tumor margin, high mature TLSTM density (mature TMTLS^high^, > 0.362 TLSs/mm^2^) was significantly correlated with improved OS (*p* < 0.0001; **Figure S5E**).

After adjustment for confounding factors, patients with mature TLSs had better OS in the multivariate analysis (HR 0.155, 95% CI 0.063 to 0.379; *p* < 0.001; **Table S2**). PD-L1 expression and pTNM were retained as independent prognostic factors in EBVaGC (HR 0.194, 95% CI 0.074 to 0.512; *p =* 0.001; HR 8.491, 95% CI 1.113 to 64.8; *p* = 0.039; **Table S2**). Collectively, our data suggested that mature TLS could serve as a precise prognostic biomarker for OS in EBVaGC patients.

**Figure 4:**
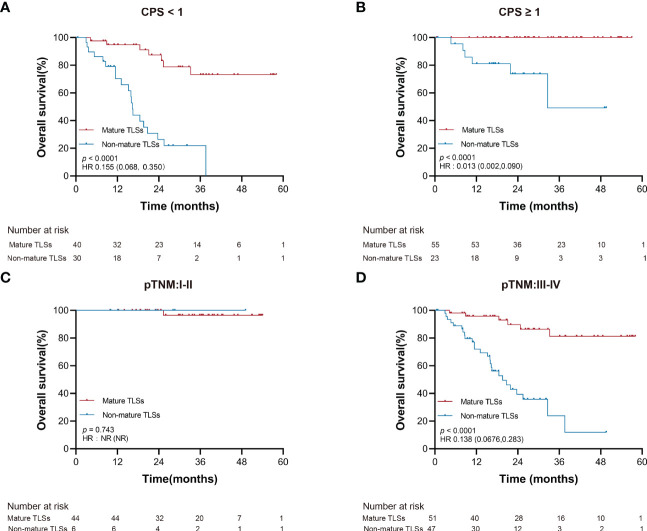
Overall survival stratified by clinicopathological risk factors. **(A, B)** PD-L1 expression (CPS). **(C, D)** pTNM. EBVaGC, Epstein-Barr virus associated gastric cancer; PD-L1, programmed death-ligand 1; CPS, the combined positive score; pTNM, pathologic tumor-node-metastasis.

Furthermore, the TLSs maturation affecting the OS of EBVaGC patients was stratified according to PD-L1 expression and pTNM. As shown in **Figure 4**, the mature TLSs were associated with better survival in patients in the subgroup of pTNM: III-IV, CPS < 1 as well as ≥1 (all *p* < 0.0001).

### TLSs as a potential biomarker for therapy responsiveness in EBVaGC

**Figure 5:**
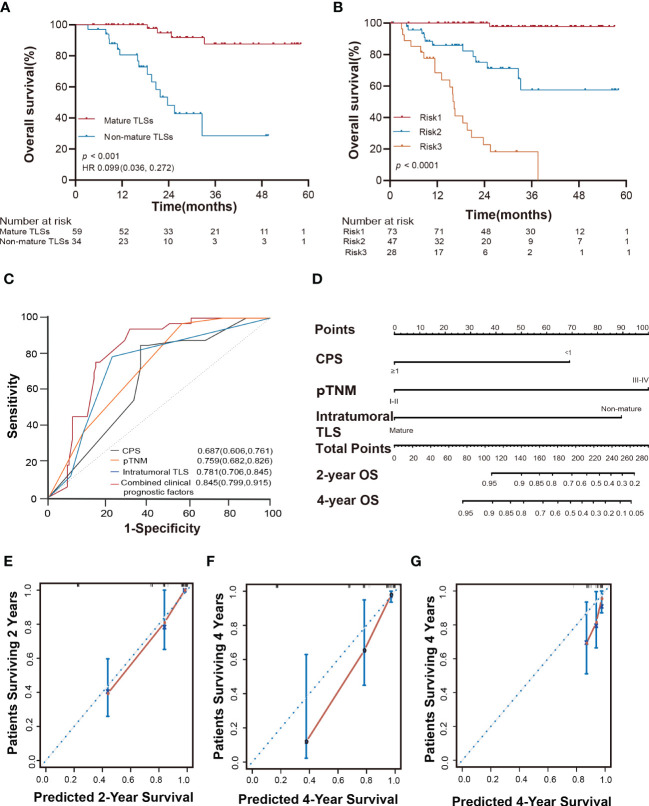
Intratumoral TLSs are associated with therapeutic response, survival risk, prognostic accuracy, and probability of overall survival in EBVaGC patients. **(A)** Kaplan-Meier curves showed different responses to adjuvant chemotherapy in different subgroups of patients in the SYSUCC cohort (log-rank test). **(B)** Kaplan-Meier curves showed that the three risk factors successfully stratified risk for the predicted survival of patients with EBVaGC in the SYSUCC cohort (log-rank test). **(C)** ROC curves compare the prognostic accuracy of the combined risk factors and any single independent risk factor in EBVaGC patients. Risk1: presence of at most one risk factors; Risk2: presence of two risk factors; Risk3: presence of three risk factors. **(D)** Nomogram for predicting the overall survival of EBVaGC patients with the independent factors. **(E, F)** The calibration curve for predicting patient overall survival at 2 years and 4 years in the SYSUCC cohort. **(G)** The calibration curve for predicting patient overall survival at 4 years of the validation cohort. EBVaGC, Epstein-Barr virus associated gastric cancer; ROC, receiver operator characteristic.

Adjuvant chemotherapy is a standard treatment for patients with advanced gastrointestinal cancer (32). In this study, we noted that the mature TLSs group could have a better OS than the non-mature TLSs group after adjuvant chemotherapy (*p* < 0.001, **Figure 5A**). Among the four patients treated with anti-PD1, partial response (PR), stable disease (SD), and progressive disease (PD) were observed in 1, 1, and 2 cases, respectively. Interestingly, the 2 PR/SD patients were in mature TLS group, while the other 2 PD patients were in the non-mature TLS group.

### Survival risk and prognostic accuracy according to different risk factors

To accurately evaluate the survival risk for EBVaGC, according to the results of the univariate and multivariate analyses, we classified the patients into three subtypes based on three prognostic factors namely intratumoral TLSs maturation, pTNM, and PD-L1 expression. Risk 1 was defined as the presence of any risk factor, risk 2 as the presence of any two risk factors, and risk 3 as the presence of all three risk factors. This could significantly stratify risk (low, intermediate, and high) for OS (*p* < 0.0001; **Figure 5B**) in the current study. Furthermore, the combined three clinicopathological risk factors also showed significantly higher prognostic accuracy than any independent clinical risk factor (AUC = 0.845, 95% CI: 0.799 to 0.915; **Figure 5C**).

### Nomogram predicts the OS probability for EBVaGC patients

To predict the absolute OS probability for each individual, we then constructed a nomogram based on the multivariate Cox model. The C-index in the SYSUCC cohort was 0.862 (95% CI: 0.816 to 0.907), which was superior to that of the C-index of the pTNM stage (0.792, 95% CI: 0.734 to 0.850; **Figure 5D**). The nomogram C-index in the validation cohort was 0.682 (95% CI: 0.553 to 0.812), superior to the C-index of the pTNM stage (0.583, 95% CI: 0.44 to 0.726). The nomogram calibration curve of OS at 2 and 4 years showed optimal agreement between the predicted and actual observations (**Figures 5E-G**). We also provide a web-based tool to predict the survival time for each patient (https://linwp.shinyapps.io/DynamicNomogramPredictingOS/).

### The profiles of somatic aberrations and immune infiltration in EBVaGC patients

**Figure 6:**
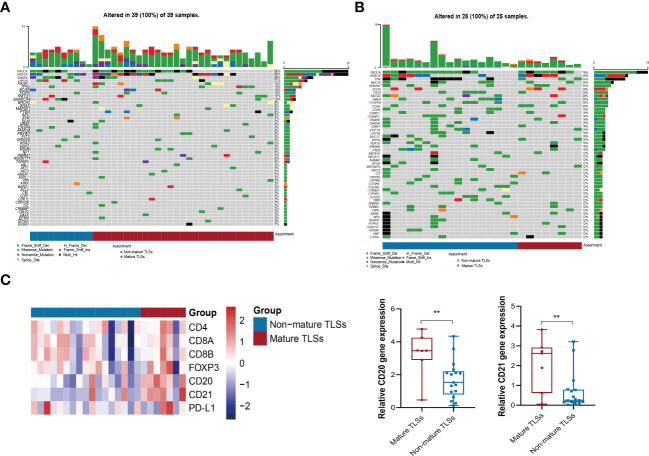
Mutation spectrum and the expression of TLS markers and PD-L1. **(A, B)** Waterfall plots show the mutation distribution of the top 50 most frequently mutated genes in the SYSUCC and TCGA cohorts. **(C)** The expression of TLS marker and PD-L1 between mature TLS and non-mature TLS groups among TCGA patients. *p* < 0.01**. TLS, tertiary lymphoid structures.

To further investigate the genomic alterations in EBVaGC, targeting NGS was performed in 29 mature TLSs cases and 10 non-mature TLSs cases in the SYSUCC cohort. Our data showed that *PIK3A* and *ARID1A* were most frequently mutated, consistent with the molecular profile described in the TCGA database (**Figures 6A, B**) (5). The most differentially mutated genes between non-mature TLSs and mature TLSs groups were *SMAD4* (50% *v* 17%), *LRP1B* (40% *v* 14%), *PIK3R1* (0% *v* 21%), and *SMARCA4 (*30% *v* 3%; *p* < 0.05; **Table S3**). Notably, *PIK3R1*, *TP53*, *SMARCA4*, and *EGFR* were identified as newly mutated genes, as these genes were among the top 10 mutated genes in EBVaGC from the SYSUCC cohort, and they were not mutated in EBVaGC from the TCGA database (**Table S4**).

To characterize the immune microenvironment of EBVaGC tumors, we also analyzed the expression of TLS markers and PD-L1 in the TCGA EBVaGC database (**Figures 6C** and **S6**). Consistent with our multiplex immunofluorescence data, the expression of CD20 and CD21 markers in the mature TLSs group was significantly higher than that in the non-mature TLSs (**Figure 6C**). To decipher the immune landscape of the two groups, the immune cell-specific marker genes were evaluated and are displayed in the corresponding heatmap in **Figure S6C**. Additionally, plasmacytoid dendritic cells and gamma delta T cells were significantly enriched in the mature TLS groups (**Figure S6C**).

## Discussion

In the two-center study, we investigated the spatial distribution, density, and TLS maturation, as well as its clinicopathological feature in EBVaGC patients. Our results showed that mature TLS was associated with a favorable outcome and response to chemotherapy in EBVaGC patients. Furthermore, the model combining three independent risk factors (intratumoral TLS maturation, pTNM, and PD-L1 expression) could significantly stratify the survival risk, and the corresponding nomogram could predict the probability of OS efficiently. Moreover, our study presented the molecular characteristics and immune landscape of EBVaGC patients. Our study revealed important information on adaptive immunity and suggested that TLSs maturation may serve as an ideal biomarker for tumor response to chemotherapy.

According to the distinctive molecular profiles, EBVaGC is a potential responder to chemotherapy and immunotherapy. Intratumoral TLSs were correlated with favorable clinical outcomes because the TLS-existing microenvironments are associated with an efficient adaptive immune response (33). Our study highlights that intratumoral mature TLS was a favorable prognostic factor for OS in two cohorts of EBVaGC patients. Besides, peritumoral mature TLSs were also associated with favorable OS in the SYSUCC cohort. Moreover, our study showed that mature TLSs were significantly associated with a good response to adjuvant chemotherapy in EBVaGC patients. More interestingly, EBVaGC patients with mature TLSs may be more sensitive to PD-1 inhibitors, suggesting that the mature TLSs may represent an effective antitumor environment.

B cells in mature TLSs can undergo somatic hypermutation and class switch recombination to generate effector and memory B cells (34). Activated B cells can ‘educate’ T cells by presenting tumor antigens, enabling the T cells to target tumor cells effectively, whereas T cells in non-mature TLSs tumors may have a dysfunctional molecular phenotype (34–36). Our multiplex immunofluorescence results also showed that the adaptive immune system (CD4^+^ T cells, CD8^+^ T cells, CD20^+^ B cells, and CD21^+^ FDCs) were more common in mature TLSs compared with non-mature TLSs. CD20^+^ B cells and CD21^+^ FDCs were also enriched in the RNA-seq data from the TCGA. The analysis of immune infiltration further showed that plasmacytoid dendritic cells (pDCs) were enriched in the mature TLS group, which specifically produce type 1 interferon (IFN) to elicit anti-viral programs and modulate immunity through effects on antigen-presenting cells and T cells (37, 38). We also found that the expression of *MAF and CD200* (markers for follicular helper T [Tfh] cells) in the TGCA RNA-seq data were significantly higher in mature TLSs compared to those of non-mature TLSs. Tfh cells, an essential constituent of TLSs, promote the germinal center formation and regulate germinal center B cell differentiation to shape the anti-tumor environment (39, 40). In summary, the mature TLSs could exert an anti-tumor immune response by coordinating both cellular and humoral responses, thus serving as a potential biomarker of chemotherapy and immunotherapy response in EBVaGC patients.

Comprehensive molecular alterations hold the promise to develop novel avenues for precision diagnostics and therapeutics. In EBVaGCs, *PIK3A* and *ARID1A* have been reported as frequently mutated genes in both previous studies and TCGA data (5). Interestingly, we found that mutation of *SMARCA4* was less frequent in EBVaGC with mature TLSs. *SMARCA4*-altered GC had intratumoral heterogeneity and histomorphological diversity (41). *SMARCA4* can also modulate the *MYC*-related enhancer and promoter in EBV-infected B cells, indicating that *SMARCA4* alterations may be associated with aggressive EBVaGC (42). We also aim to investigate the differences in intratumoral B cells between *SMARCA4* mutant and non-mutant EBVaGC patients in our future work. By characterizing the mutational landscape of EBVaGC with mature TLSs and non-mature TLSs, we believe that our work may provide supporting evidence for elucidating the relevant molecular mechanisms of EBVaGC.

The retrospective nature of this research could be its main limitation. In addition, the different timeline nature of the two cohorts may be considered as a limitation of this study. However, the study was strengthened by the fact that the cohorts from different timelines had yielded similar results. Furthermore, NGS analyses revealed four newly mutated genes in EBVaGC from the SYSUCC cohort. Further molecular biology experiments are needed to gain important insights into their functions and underlying machinery. Nevertheless, our results provide the most compelling and comprehensive evidence to date for the role of TLSs in the prognosis and treatment of EBVaGC.

In summary, our findings revealed that mature TLS was a powerful and favorable prognostic factor for the survival of EBVaGC patients and could potentially identify patients who could benefit from adjuvant chemotherapy and immunotherapy. Furthermore, the three independent risk factors (intratumoral TLSs maturation, pTNM, and PD-L1 expression) provided the highest accuracy in predicting the risk and possibility of survival. The mutation profile also provides significant value for elucidating the relevant molecular mechanisms of EBVaGC. The findings from this study yielded new insights into identifying potential markers for cancer therapy and evidence for improving the survival of EBVaGC patients.

## Data availability statement

The data presented in the study are deposited in the Research Data Deposit (www.researchdata.org.cn), accession number RDDB2022391348.

## Ethics statement

The studies involving human participants were reviewed and approved by Institutional Medical Ethics Committee of Sun Yat-Sen University Cancer Center and Chinese Academy of Medical Sciences and Peking Union Medical College. The patients/participants provided their written informed consent to participate in this study.

## Author contributions

Y-XY, Y-HL and M-YC designed the study. Y-XY, Y-HL, X-LW, L-YX, C-YH, B-Z W and C-FH collected the data. Y-XY and W-PL analyzed the data. R-CN, J-WC, J-LL, J-JX, JZ, J-PY and DX provided advice and oversaw a portion of the work. Y-XY and M-YC drafted the manuscript. M-YC critically reviewed the manuscript. All authors contributed to the article and approved the submitted version.
