# Supplementary material for: Impact of mature tertiary lymphoid structures on prognosis and therapeutic response of Epstein-Barr virus-associated gastric cancer patients
Source: Front Immunol. 2022 Dec 14;13:973085. doi: 10.3389/fimmu.2022.973085 (PMC9794571; doi:10.3389/fimmu.2022.973085)
Supplement: Supplementary file 1 [file DataSheet_1.pdf]

Supplemental data

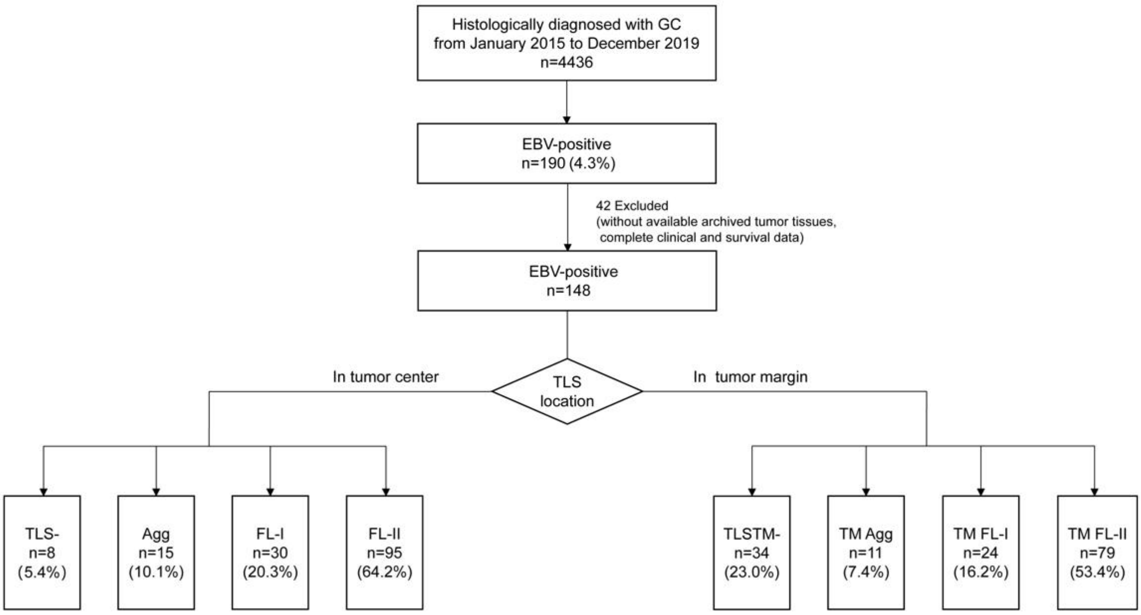

Figure S1. Study flow chart in the SYSUCC cohort. EBV-positive patients meeting the criteria were selected from the continuous GC cohort and TLSs were assessed according to location and maturation. EBV, Epstein-Barr virus; GC, gastric cancer; TLSs, tertiary lymphoid structures; TLS-, without any TLSs in tumor center; TLSTM-, without any TLSs in tumor margin; Agg, aggregates in tumor center; TM Agg, aggregates in tumor margin; FL-I, primary follicles; FL-II, secondary follicles.

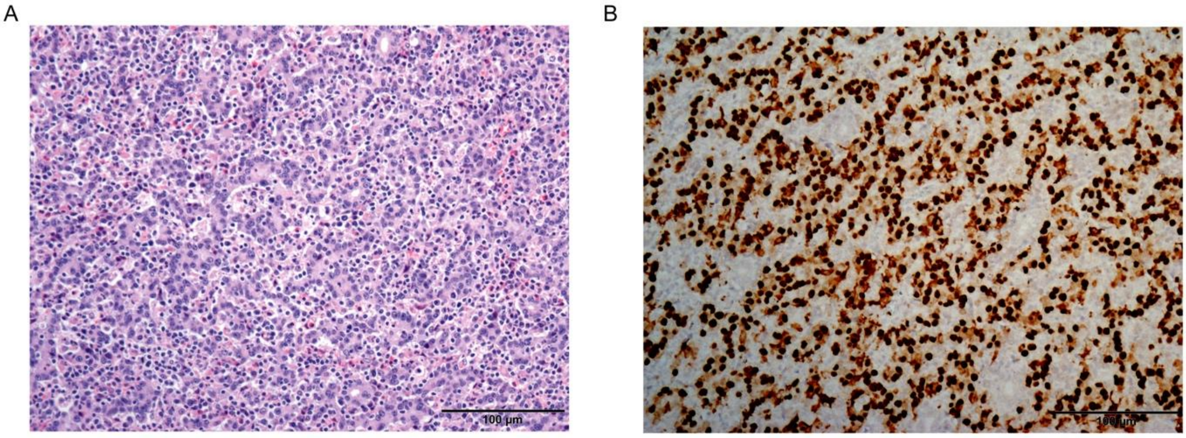

Figure S2. Examples of EBER staining morphology in EBVaGC patients. (A) Hematoxylin and eosin (H&E) slide in EBVaGC tissues; (B) The corresponding images of EBER detection. EBER-ISH, EBV-encoded RNA in situ hybridization; EBVaGC, Epstein-Barr virus associated gastric cancer.

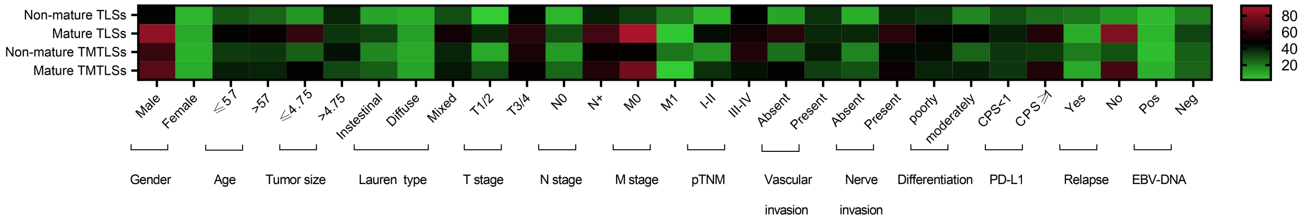

Figure S3. Heat map to describe the clinicopathological characteristics of Epstein-Barr virus associated gastric cancer patients in the SYSUCC cohort.

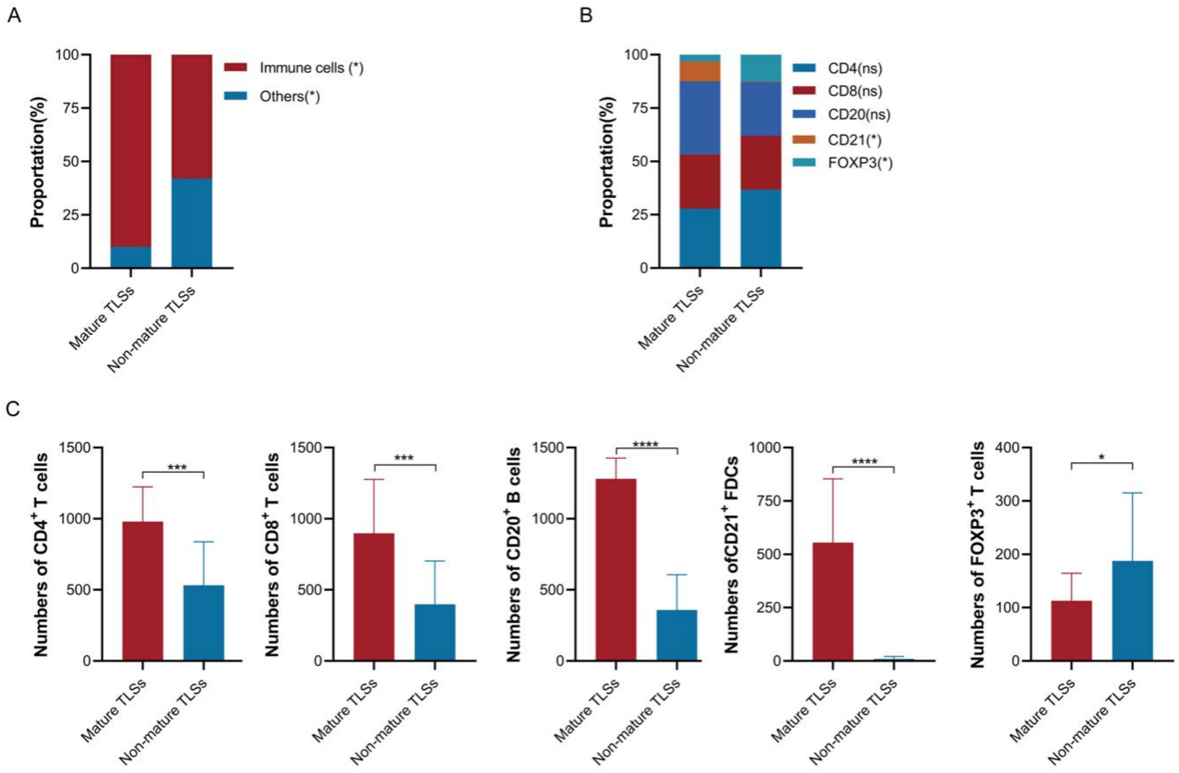

Figure S4. Quantification of the infiltrating immune cells. (A) The percentages of infiltrating immune cells in mature TLSs and non-mature TLSs. (B) The percentages of cells, describing the immune cell composition. (C) The number of infiltrating immune cells in mature TLSs or non-mature TLSs. Numbers of infiltrating immune cells were counted at fifteen random high-power fields of three slides. Magnification, x200. p < 0.05*, p < 0.01**, p < 0.001*** and p < 0.0001****. TLSs, tertiary lymphoid structures; FDCs, Follicular dendritic cells.

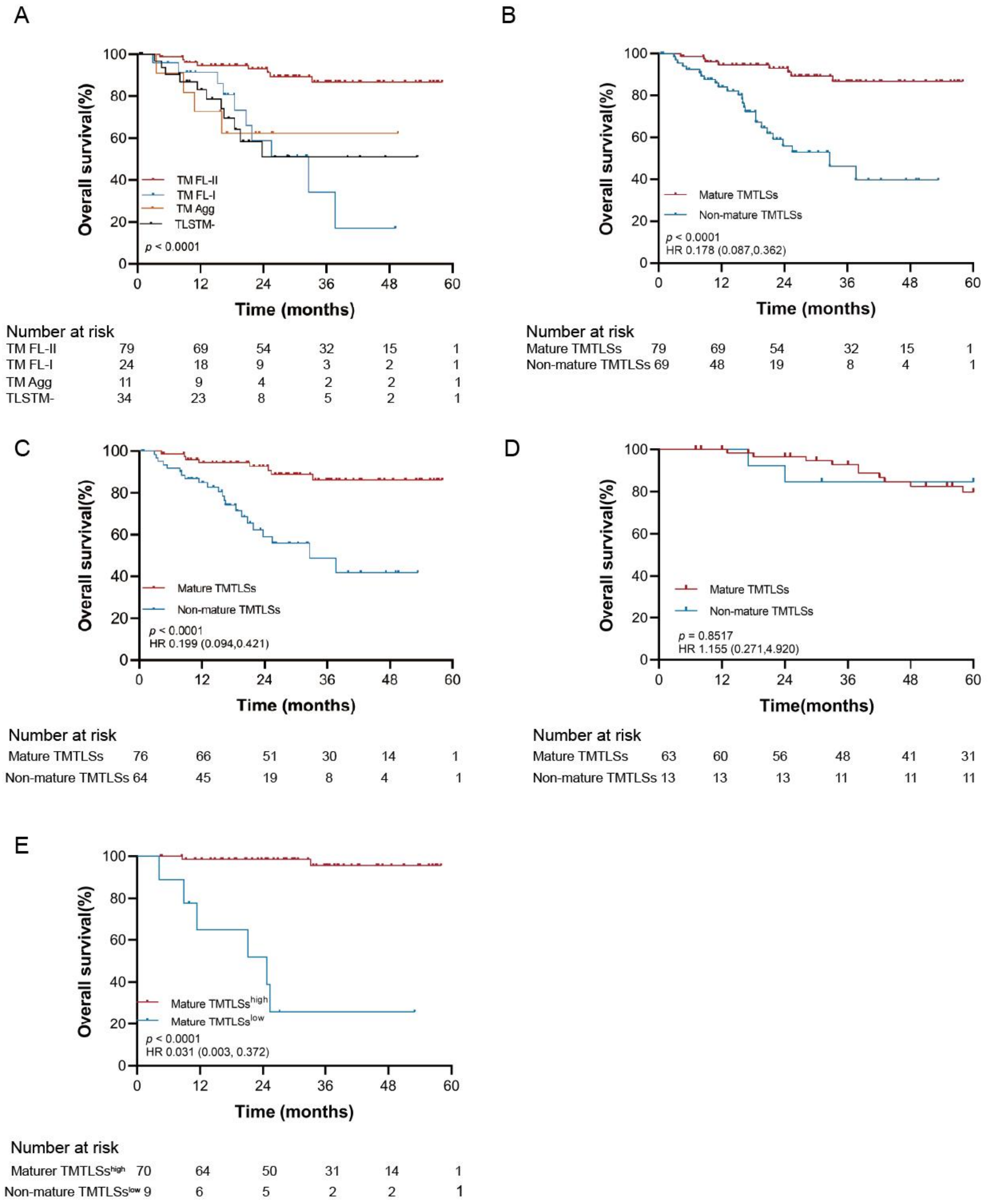

Figure S5. Kaplan-Meier estimates of overall survival according to the maturation of peritumoral TLSs (log-rank test). (A) Probability of survival of patients in TLSTM-, TM Agg, TM FL-I, and TM FL-II groups in the SYSUCC cohort. (B) Probability of survival of patients in mature TMTLSs and non-mature TMTLSs

groups in the SYSUCC cohort. (C) Probability of survival of patients in mature TMTLSs and non-mature TMTLSs groups in the SYSUCC cohort, except receiving neoadjuvant chemotherapy patients. (D) Probability of survival of patients in mature TMTLSs and non-mature TMTLSs groups in the validation cohort.

(E) Probability of survival of patients in mature $TMTLSs^{high}$ and mature $TMTLSs^{low}$ groups in the SYSUCC cohort. EBVaGC: Epstein-Barr virus associated gastric cancer; TLSs: tertiary lymphoid structures; TM Agg, aggregates in tumor margin; TM FL-I: primary follicles in tumor margin; TM FL-II: secondary follicles in tumor margin; Mature TMTLSs: known as FL-II in tumor margin; Non-mature TMTLSs: including TLSTM-, TM Agg, TM FL-I; Mature $TMTLSs^{high}$: high density of mature TLSs in tumor margin; Mature $TMTLSs^{low}$: low density of mature TLSs in tumor margin.

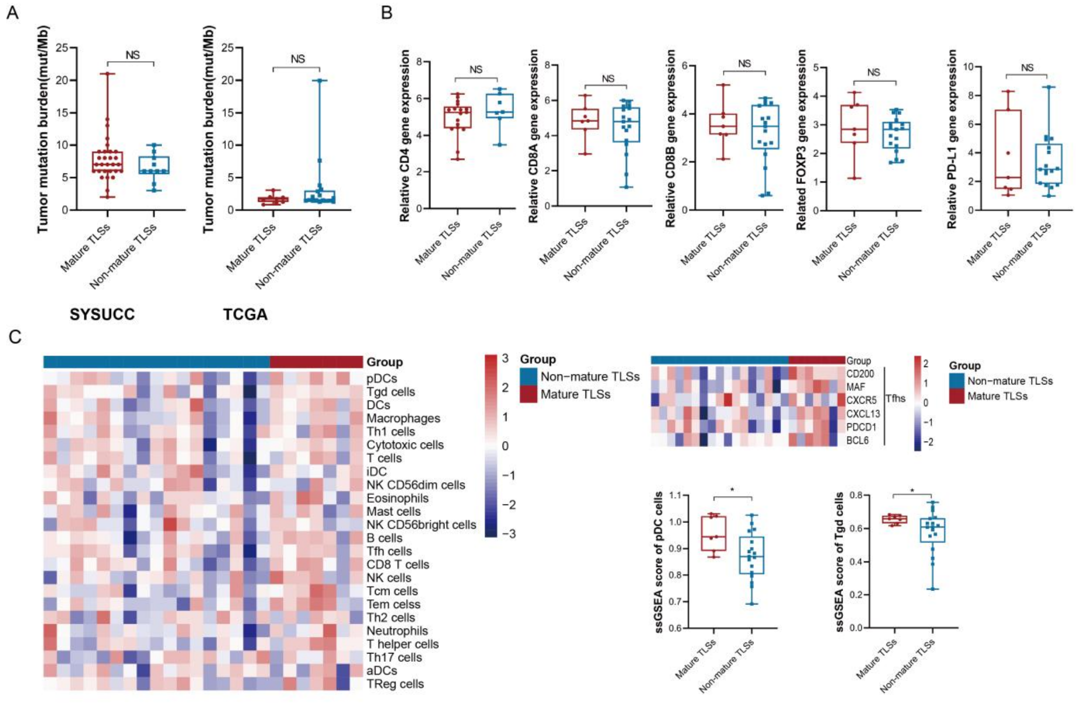

Figure S6. Immune infiltration profile and TMB of EBVaGC patients. (A) Tumor mutation burden in mature TLSs or non-mature TLSs between the SYSUCC and TCGA cohorts. (B) The expression level of some TLS markers and PD-L1 in mature TLSs or non-mature TLSs from the TCGA cohort. (C) The immune infiltration patterns in mature TLSs or non-mature TLSs from the TCGA cohorts. $p < 0.05$*. SYSUCC, Sun Yat-sen University Center; Tfhs: T follicular helper cells; pDC, plasmacytoid dendritic cells; Tgd cells, T gamma delta cells.

Table S1. Correlation between the maturation of TLS and clinico-pathological features in EBV-positive gastric cancer in the the validation cohort

Characteristics	Available number	Tumor center			Tumor margin		
		Non-mature TLSs	Mature TLSs	*P* value[a]	Non-mature TMTLSs	Mature TMTLSs	*P* value[a]
Gender	76			0.922			0.970
Male	67 (88.2%)	36 (53.7%)	31 (46.%)		12 (17.9%)	55 (82.1%)	
Female	9 (11.8%)	4 (44.4%)	5 (55.6%)		1 (11.1%)	8 (88.9%)	
Age (years)	76			0.981			0.261
≤ 57.0[b]	40 (52.6%)	21 (52.5%)	19 (47.5%)		5 (12.5%)	35 (92.1%)	
> 57.0	36 (47.4%)	19 (52.8%)	17 (47.2%)		8 (22.2%)	28 (73.7%)	
Tumor size(cm)	76			0.168			0.033
≤ 4.0[c]	38 (50.0%)	17 (44.7%)	21 (55.3%)		3 (7.9%)	35 (92.1%)	
> 4.0	38 (50.0%)	23 (60.5%）	15 (39.5%)		10 (26.3%)	28 (73.7%)	
Lauren type	76			0.126			0.862
Intestinal	12 (15.8%)	5 (41.7%)	7 (58.3%)		2 (16.7%)	10 (83.3%)	
Diffuse	36 (47.4%)	16 (44.4%)	20 (55.6%)		7 (19.4%)	29 (80.6%)	

Mixed	28 (36.8%）	19 (67.9%)	9 (32.1%)		4 (14.3%)	24 (85.7%)	
T stage	76			< 0.001			0.085
T1+T2	34 (44.7%)	10 (29.4%)	24 (70.6%)		3 (8.8%)	31(91.2%)	
T3+T4	42 (55.3%)	30 (71.4%)	12 (28.6%)		10 (23.8%)	32(76.2%)	
N stage	76			< 0.001			0.225
N0	41 (53.9%)	15 (36.6%)	26 (63.4%)		9 (22.0%)	32 (78.0%)	
N+	35 (46.1%)	25(71.4%)	10 (28.6%)		4 (11.4%)	31 (88.6%)	
M stage	76			1.000			0.647
M0	75 (98.7%)	39 (52.0%)	36 (48.0%)		13 (17.3%)	62 (82.7%)	
M1	1 (1.3%)	1 (100.0%)	0 (0.0%)		0 (0.0%)	1(100.0%)	
pTNM	76			< 0.001			0.353
I-II	50 (65.8%)	19 (38.0%)	31 (62.0%)		10 (20.0%)	40 (80.0%)	
III-IV	26 (34.2%)	21 (80.8%)	5 (19.2%)		3 (11.5%)	23 (88.5%)	
Vascular invasion	76			0.069			0.694
Absent	49 (64.5%)	22 (44.9%)	27 (55.1%)		9 (18.4%)	40 (81.6%)	
Present	27 (35.5%)	18 (66.7%)	9 (33.3%)		4 (14.8%)	23 (85.2%)	
Neural invasion	76			0.498			0.841

Absent	37 (48.7%)	18 (48.6%)	19 (51.4%)		6 (16.2%)	31 (83.8%)	
Present	39 (51.3%)	22 (56.4%)	17 (43.6%)		7 (17.9%)	32 (82.1%)	
Differentiation	76			0.601			0.435
Poorly	73 (96.1%)	39 (53.4%)	34 (46.6%)		12 (16.4%)	61 (83.8%)	
Moderately	3 (4.1%)	1 (33.3%)	2 (66.7%)		1 (33.3%)	2 (66.7%)	
PD-L1 expression	76			0.841			0.823
CPS < 1	13 (17.1%)	7 (53.8%)	6 (46.2%)		3 (23.1%)	10 (76.9%)	
CPS ≥ 1	63 (82.9%))	32 (50.8%)	31 (49.2%)		10 (15.9%)	53 (84.1%)	

[a]Chi-square test; [b]Median age; [c]Median size. TLS, tertiary lymphoid structure;

EBV, Epstein-Barr virus; PD-L1, programmed death-ligand 1; CPS, the combined positive score.

Table S2. Univariate and multivariate analyses of patients with EBV-positive gastric cancer of the SYSUCC cohort

Characteristics[a]	*P* value	Hazard Ratio(95%CI)
**Univariate analysis**		
Gender (Female vs. Male)	0.774	1.166 (0.409, 3.322)
Age (>57.0[b] vs. ≤ 57.0)	0.912	1.039 (0.525, 2.058)
Tumor size(cm)(>4.75[c] vs. ≤ 4.75)	< 0.001	5.046 (2.179, 11.687)
Lauren type		
Intestinal		
Diffuse	0.061	2.757 (0.956, 7.949)
Mixed	0.300	1.636 (0.645, 4.151)
T stage (T3+T4 vs. T1+T2)	0.007	15.456 (2.103, 113.604)
N stage (N+ vs. N0)	0.029	36.974 (1.435, 952.626)
M stage (M1 vs. M0)	<0.001	7.923 (3.810, 16.475)
pTNM (III-IV vs. I-II)	0.002	23.511 (3.208, 172.303)
Vascular invasion (Present vs. absent)	<0.001	6.885 (2.394, 19.801)
Neural invasion (Present vs. absent)	0.005	7.862 (1.869, 33.071)
Differentiation (Moderately vs. poorly)	0.26	0.665 (0.327, 1.352)
PD-L1 expression (CPS ≥ 1 vs. CPS < 1)	< 0.001	0.155 (0.064, 0.377)
EBV-DNA (Positive vs. negative)	0.461	0.456 (0.057, 3.673)
Intratumoral TLS (TLS FL-II^{+} vs. TLS FL-II^{-})	< 0.001	0.086 (0.036, 0.202)
Peritumoral TLS (TLSTM FL-II^{+} vs. TLSTM FL-II^{-})	< 0.001	0.166 (0.074, 0.374)
**Multivariate analysis**		
Intratumoral TLS (TLS FL-II^{+} vs. TLS FL-II^{-})	< 0.001	0.155 (0.063, 0.379)
PD-L1 expression (CPS ≥ 1 vs. CPS < 1)	0.001	0.194 (0.074, 0.512)
pTNM (III-IV vs. I-II)	0.039	8.491 (1.113, 64.8)

[a]The analyses were performed with the use of Cox proportional-hazards regression; [b]Median age; [c]Median size. EBV, Epstein-Barr virus; PD-L1, programmed death-ligand 1; CPS, the combined positive score; TLS, tertiary lymphoid structures.

Table S3 The mutation distribution of the top 50 most frequently mutated genes from the SYSUCC and TCGA cohorts.

	SYSUCC cohort					TCGA cohort					
	Non-mature TLSs (n=10)		Mature TLSs (n=29)				Non-mature TLSs (n=17)		Mature TLSs (n=8)		
Gene	Number with alterations	Percentage with alterations	Number with alterations	Percentage with alterations	*P* *value*	Gene	Number with alterations	Percentage with alterations	Number with alteration s	Percentag e with alterations	*P* *value*
PIK3CA	6	60%	16	55%	1	PIK3CA	12	71%	7	88%	0.624
ARID1A	5	50%	17	57%	0.721	ARID1A	7	41%	5	63%	0.411
SMAD4	5	50%	5	17%	0.087	TTN	8	47%	3	38%	1
LRP1B	4	40%	4	14%	0.167	MUC16	5	29%	2	25%	1
PIK3R1	0	0	6	21%	0.308	AHNAK2	4	24%	2	25%	1
TP53	1	10%	5	17%	1	BCOR	2	12%	3	38%	0.283
BCOR	1	10%	3	10%	1	FAT4	4	24%	1	13%	1
EGFR	1	10%	3	10%	1	KMT2D	4	24%	1	13%	1
KMT2D	1	10%	3	10%	1	NBEA	5	29%	0	0	0.14
SMARCA4	3	30%	1	3%	0.045	PCDH10	4	24%	1	13%	1
BRCA2	1	10%	2	7%	1	TCHH	2	12%	3	38%	0.283
GNAS	0	0	3	10%	0.556	CDH9	4	24%	0	0	0.269
MAP2K1	1	10%	2	7%	1	CSMD1	3	18%	1	13%	1
PTEN	0	0	3	10%	0.556	CSRNP3	3	18%	1	13%	1
APC	0	0	2	7%	1	DNAH5	3	18%	1	13%	1
ATM	0	0	2	7%	1	DNAH8	3	18%	1	13%	1
BLM	1	10%	1	3%	0.452	GRIK1	3	18%	1	13%	1
BRAF	0	0	2	7%	1	IGSF10	3	18%	1	13%	1
CASP8	1	10%	1	3%	0.452	LRP1B	4	24%	0	0	0.269
FANCM	1	10%	1	3%	0.452	MUC17	4	24%	0	0	0.269

FBXW7	0	0	2	7%	1	MYH2	2	12%	2	25%	0.57
FLT3	1	10%	1	3%	0.452	NEK10	3	18%	1	13%	1
GRIN2A	1	10%	1	3%	0.452	PRDM9	3	18%	1	13%	1
KRAS	0	0	2	7%	1	PTEN	3	18%	1	13%	1
MSH2	1	10%	1	3%	0.452	ABCA10	2	12%	1	13%	1
MSH6	0	0	2	7%	1	ABCA13	3	18%	0	0	0.527
NF1	0	0	2	7%	1	AHNAK	3	18%	0	0	0.527
PBRM1	0	0	2	7%	1	APOB	3	18%	0	0	0.527
RUNX1T1	0	0	2	7%	1	ARFGEF2	2	12%	1	13%	1
TGFBR2	1	10%	1	3%	0.452	BIRC6	3	18%	0	0	0.527
ABL1	0	0	1	3%	1	CIT	1	6%	2	25%	0.231
AKT1	0	0	1	3%	1	CMYA5	3	18%	0	0	0.527
AKT3	0	0	1	3%	1	CNTN6	3	18%	0	0	0.527
AMER1	0	0	1	3%	1	COL4A4	3	18%	0	0	0.527
ASXL1	0	0	1	3%	1	COL6A3	3	18%	0	0	0.527
ATR	0	0	1	3%	1	CTNNB1	3	18%	0	0	0.527
ATRX	1	10%	0	0	0.256	CYP4F2	3	18%	0	0	0.527
BARD1	0	0	1	3%	1	DCHS2	3	18%	0	0	0.527
BCL2	0	0	1	3%	1	DMD	3	18%	0	0	0.527
CBL	1	10%	0	0	0.256	DNAH9	3	18%	0	0	0.527
CDH1	0	0	1	3%	1	ERBB3	2	12%	1	13%	1
CDK12	0	0	1	3%	1	FBN2	2	12%	1	13%	1
CDKN2B	0	0	1	3%	1	MBD1	2	12%	1	13%	1
CIC	0	0	1	3%	1	NEB	3	18%	0	0	0.527
CREBBP	0	0	1	3%	1	NLRP9	3	18%	0	0	0.527
CRLF2	0	0	1	3%	1	PTPRD	2	12%	1	13%	1
DAXX	0	0	1	3%	1	SCN11A	2	12%	1	13%	1
EP300	0	0	1	3%	1	USH2A	3	18%	0	0	0.527
EPHA3	0	0	1	3%	1	VWF	2	12%	1	13%	1
ERBB3	0	0	1	3%	1	ZFHX4	2	12%	1	13%	1

Table S4 Comparison of the top 10 genes in EBVaGC in SYSUCC and TCGA cohorts.

	EBVaGC in SYSUCC		EBVaGC in TCGA	
Gene	Number of case	Percentage	Number of case	Percentage
PIK3CA	22	56.41%	19	76.00%
ARID1A	22	56.41%	12	48.00%
SMAD4	10	25.64%	3	12.00%
LRP1B	8	20.51%	4	16.00%
PIK3R1	6	15.38%	0	0
TP53	6	15.38%	0	0
BCOR	4	10.26%	5	20.00%
EGFR	4	10.26%	0	0
KMT2D	4	10.26%	5	20.00%
SMARCA4	4	10.26%	0	0
